# Glucose-6-Phosphate Dehydrogenase (G6PD)-Deficient Epithelial Cells Are Less Tolerant to Infection by *Staphylococcus aureus*

**DOI:** 10.1371/journal.pone.0079566

**Published:** 2013-11-04

**Authors:** Yi-Ting Hsieh, Mei-Hui Lin, Hung-Yao Ho, Lei-Chin Chen, Chien-Cheng Chen, Jwu-Ching Shu

**Affiliations:** 1 Department of Medical Biotechnology and Laboratory Science, College of Medicine, Chang Gung University, Taoyuan, Taiwan; 2 Research Center for Pathogenic Bacteria, Chang Gung University, Taoyuan, Taiwan; 3 Healthy Aging Research Center, Chang Gung University, Taoyuan, Taiwan; 4 Department of Nutrition, I-Shou University, Kaohsiung, Taiwan; 5 Department of Biotechnology, National Kaohsiung Normal University, Kaohsiung, Taiwan; University of Iowa Carver College of Medicine, United States of America

## Abstract

Glucose-6-phosphate dehydrogenase (G6PD) is a key enzyme in the pentose phosphate pathway and provides reducing energy to all cells by maintaining redox balance. The most common clinical manifestations in patients with G6PD deficiency are neonatal jaundice and acute hemolytic anemia. The effects of microbial infection in patients with G6PD deficiency primarily relate to the hemolytic anemia caused by *Plasmodium* or viral infections and the subsequent medication that is required. We are interested in studying the impact of bacterial infection in G6PD-deficient cells. G6PD knock down A549 lung carcinoma cells, together with the common pathogen *Staphylococcus aureus*, were employed in our cell infection model. Here, we demonstrate that a lower cell viability was observed among G6PD-deficient cells when compared to scramble controls upon bacterial infection using the MTT assay. A significant increase in the intracellular ROS was detected among *S. aureus*-infected G6PD-deficient cells by observing dichlorofluorescein (DCF) intensity within cells under a fluorescence microscope and quantifying this signal using flow cytometry. The impairment of ROS removal is predicted to enhance apoptotic activity in G6PD-deficient cells, and this enhanced apoptosis was observed by annexin V/PI staining under a confocal fluorescence microscope and quantified by flow cytometry. A higher expression level of the intrinsic apoptotic initiator caspase-9, as well as the downstream effector caspase-3, was detected by Western blotting analysis of G6PD-deficient cells following bacterial infection. In conclusion, we propose that bacterial infection, perhaps the secreted *S. aureus* α-hemolysin in this case, promotes the accumulation of intracellular ROS in G6PD-deficient cells. This would trigger a stronger apoptotic activity through the intrinsic pathway thereby reducing cell viability when compared to wild type cells.

## Introduction

Glucose-6-phosphate dehydrogenase (G6PD) is the key enzyme that catalyzes the first reaction, the oxidation of glucose-6-phosphate to 6-phosphogluconolactone, in the pentose phosphate pathway, thereby providing reducing energy to all cells by maintaining the level of the reduced co-enzyme nicotinamide adenine dinucleotide phosphate (NADPH). NADPH plays an important role in maintaining the supply of reduced glutathione to counterbalance oxidant-induced oxidative stress [1]. Redox imbalance may induce cell apoptosis and necrosis, thus highlighting the role of G6PD in defending against oxidative damage [2,3]. G6PD deficiency is the most prevalent enzyme defect in humans and affects an estimated 400 million people worldwide, especially in populations historically exposed to endemic malaria [4]. The most common clinical manifestations are neonatal jaundice and acute hemolytic anemia, which is caused by the impairment of the erythrocyte’s ability to remove harmful oxidative stress triggered by exogenous agents such as drugs, infection, or fava bean ingestion [1,4]. 

Hemolytic anemia caused by infection and subsequent medication is a clinically important concern in patients with G6PD deficiency. This issue has been a primary focus for many decades in relation to efforts to understand the impact of *Plasmodium* infection (malaria) and antimalarial drugs [5,6]. Antimicrobial drug-induced hemolysis is considered the most common adverse clinical consequence of G6PD deficiency [7]. It has also been demonstrated that infections caused by certain viruses, such as hepatitis viruses (A, B, and E) and cytomegalovirus, were associated with hemolytic anemia in patients with G6PD deficiency [8,9]. Recently, it has been shown that infection by particular viruses, such as enterovirus 71, dengue virus, and coronavirus, was enhanced in G6PD-deficient cells [10–12]. 

However, the impact of bacterial infection on patients with G6PD deficiency still remains to be clarified. Most studies have focused on investigating the antibiotic-induced hemolysis after treatment for bacterial infection [7]. In addition, a case report showed that infection by *Clostridium difficile* may have triggered hemolysis and led to severe jaundice in a G6PD-deficient neonate, while another case report described hemolysis caused by *Acinetobacter baumannii* infection [13,14]. Wilmanski and colleagues demonstrated that hyperinflammation (increasing cytokine levels) caused by acute endotoxemia (induced by the injection of *Escherichia coli* lipopolysaccharide) resulted in increased mortality in G6PD-deficient mice [15]. Several studies also indicated that G6PD deficiency in leukocytes can result in chronic granulomatous disease (CGD) and possibly alter the host defense mechanisms for bacterial infections [16–18]. Thus far, the impact of bacterial infection on patients with G6PD deficiency has been found to primarily affect the blood cells, leading to hemolysis or immune weakness based upon the above studies.

Bacterial infection or treatment with septic plasma might induce mitochondrial dysfunction by the accumulation of reactive oxygen species (ROS) and nitric oxide radical (NO^．^) in lymphocytes or epithelial cells leading to cell apoptosis [19–21]. Therefore, we propose that cells with G6PD deficiency may be less tolerant to the oxidative stress caused by bacterial infection. In the present study, we investigate the direct impact of bacterial infection on G6PD-deficient epithelial cells using *Staphylococcus aureus* as a model pathogen. *S. aureus*, which has long been recognized as a major cause of healthcare-associated infections, can result in sepsis and septic shock leading to vascular damage and multiple organ failure. Vancomycin is one of the primary choices to treat infections resulting from multidrug-resistant *S. aureus*, including methicillin-resistant *S. aureus* (MRSA). Our previous study demonstrated that the vancomycin-treated vancomycin-resistant *S. aureus* (VRSA) strain did enhance cytotoxicity through the activation of σ^B^ and alternation of virulence expression [22]. Whether such enhancement is even stronger in G6PD-deficient cells was also investigated in this study. 

## Materials and Methods

### Bacterial strain and growth condition

The vancomycin-resistant *S. aureus* strain SJC1200 was generated by introducing a vancomycin resistance-carrying plasmid (pG1546) into strain ATCC 12598 as described previously [23]. Briefly, the *P*_*R*_ *vanRSP*_*H*_ *HAXP*_*Y*_ *vanYP*_*Z*_ *vanZ* gene cluster (the *van* operon within Tn*1546*) in *E. faecalis* HIP12467 was amplified and then cloned into pGHL6 from which the *luxAB* gene was removed to generate pG1546. All bacterial strains were routinely cultured at 37°C with the specific required antibiotics (Sigma) in BHI broth or on agar plates.

### Cell culture and bacterial infection

A lung carcinoma cell line (A549) obtained from the American Type Culture Collection (ATCC) and derivatives of this cell line were used in the present study. The stable G6PD-knockdown cell line, A549-5.20, and the control cell line transfected with pCI-neo vector only, A549-5S-5, were generated, approved and kindly given by Dr. Hung-Yao Ho [11]. Briefly, G6PD-RNAi plasmids were generated by the ligation of complementary oligonucleotides into the pCI-neo mammalian expression vector followed by transfection into A549 cells to generate A549-5.20. The cells were cultured in DMEM (Gibco BRL) supplemented with 10% fetal calf serum, penicillin (100 U/ml), and streptomycin (100 U/ml) at 37°C in a humidified atmosphere of air and 5% CO_2_. 

Before infection, overnight culture of the *S. aureus* strain SJC1200 was diluted back to an O.D._A600_ of 0.2 and sub-cultured in 10 ml BHI broth without any antibiotic at 37°C until the O.D._A600_=0.6. Bacterial infection of cells was performed by inoculating with SJC1200 (at a multiplicity of infection of 100; MOI=100) in the absence or presence of vancomycin (32 μg/ml). Under specified circumstances, the function of α-hemolysin secreted by *S. aureus* was inhibited by adding Oroxylin A (2 μg/ml; Sigma) [24], and the infected cells were cultured at 37°C in a humidified atmosphere of air and 5% CO_2_ for further use. For samples that were to be observed using a microscope, sterile glass cover slips were inserted into each of the wells in advance. 

### Bacterial killing kinetic assay

The VRSA strain SJC1200 was grown overnight at 37°C with shaking in BHI broth. The next day, the bacterial culture was diluted (1/200) in fresh BHI broth with or without vancomycin (32 μg/ml) and then incubated at 37°C with shaking. At 0, 3, 6, 9, 12, and 24 h, the bacteria were plated on agar for enumeration of the surviving cfus. Time-kill curves were then constructed by plotting the mean colony counts (log_10_ CFU/ml) versus time from three experiments. 

### Determination of cell viability

Cell viability tests were performed using the MTT assay with the cell proliferation reagent MTT (3-[4,5-dimethylthiazol-2-yl]-2,5-diphenyl tetrazolium bromide; Sigma), as described previously [25]. The MTT tetrazolium ring is cleaved only by active mitochondria, yielding purple formazan crystals whose amount directly correlates with the viable cell count. At 16 h post-infection, 10 μl of a 5 mg/ml MTT solution was added into each well, and the plates were incubated at 37°C for 2.5 h. The purple formazan crystals were dissolved by adding 100 μl of MTT solubilization solution (Sigma), and the absorbance at A_570_ was spectrophotometrically measured with a reference wavelength of A_690_. The results were expressed as the percent absorbance of each experimental well versus the well containing untreated cells. Three wells per experimental condition were counted in three independent experiments.

### Detection of reactive oxygen species (ROS)

The formation of intracellular ROS was visualized by detecting dichlorofluorescein (DCF) derived from the oxidation of dihydrodichlorofluorescein (H_2_DCF) as previously described [12]. At 12 h post-infection, 5 μM dichlorofluorescein diacetate (H_2_DCFDA; Invitrogen) was added to each well at 37°C for 20 min and examined under a fluorescence microscope. 

Quantification of ROS formation was performed by flow cytometry. Cells were treated as described above followed by two washes in PBS and trypsin treatment. Flow cytometric and data analyses were performed as described elsewhere [12]. The mean fluorescence intensity (MFI) of the DCF channel was determined using CellQuest Pro software (Becton Dickinson). The results were expressed as the fold change of the percentage increase in DCF channel. 

### Assessment of cell apoptosis

Cell apoptosis/necrosis was visualized by annexin V-FITC/propidium iodide (PI) staining with a commercial apoptosis detection kit (BioVision) by microscopy according to the manufacturer’s instructions, as well as analysis by flow cytometry. Phosphatidylserine translocation in apoptotic cells was stained with annexin V-FITC showing green fluorescence, whereas the nucleic acids in dead (necrosis) cells was stained with PI emitting red fluorescence. DAPI was employed to stain the cell nucleus, producing blue fluorescence. At 16 h post-infection, cells were washed three times with PBS. For microscopy, 1× binding buffer was added to each well together with DAPI, Annexin V-FITC, and PI (5 μl of each) for 5 min. Cover slips were removed and observed by confocal microscopy. 

For flow cytometry analysis, cells were trypsinized following a wash in PBS and resuspended in 1× binding buffer. Five min after the addition of Annexin V-FITC and PI (5 μl of each), flow cytometry analysis was performed using a FACSCalilbur (BD Bioscience). The cells were excited with the 488 nm laser and green apoptotic cells were detected at 530 nm, while red necrosis cells were detected at 620 nm. No fluorescence could be detected in live cells, and cells showing both green and red fluorescence were considered to be in late apoptosis. 

Biochemical markers of apoptosis were also detected by Western blotting analysis. The cells were lysed in lysis buffer described elsewhere and the protein concentration of the lysate was determined by the Bradford method [26]. After resolving the protein lysates by SDS-PAGE, the samples were transferred to nitrocellulose membranes and blocked with 5% milk in TBS containing 0.1% Tween 20 (TBST). The membranes were then probed with 1:1000 primary antibody (mouse multiclonal; Sigma) caspase-3, caspase-8, caspase-9 or GAPDH at 4°C overnight. After washing in TBST, membranes were probed with 1:5000 secondary horse anti-mouse HRP-linked antibody (Sigma) for 1 h and developed using ECL reagents (Bioman). The blots were photographed using an X-OMAT 2000 (Kodak) film processor. 

### Statistical analysis

A Student’s *t*-test and non-parametric tests were used to analyze the experimental data and to compare means. *P*-values of less than 0.05 were considered statistically significant.

## Results

### G6PD-deficient cells exhibit a higher cytotoxic effect than scramble cells after *S. aureus* infection

**Figure 1:**
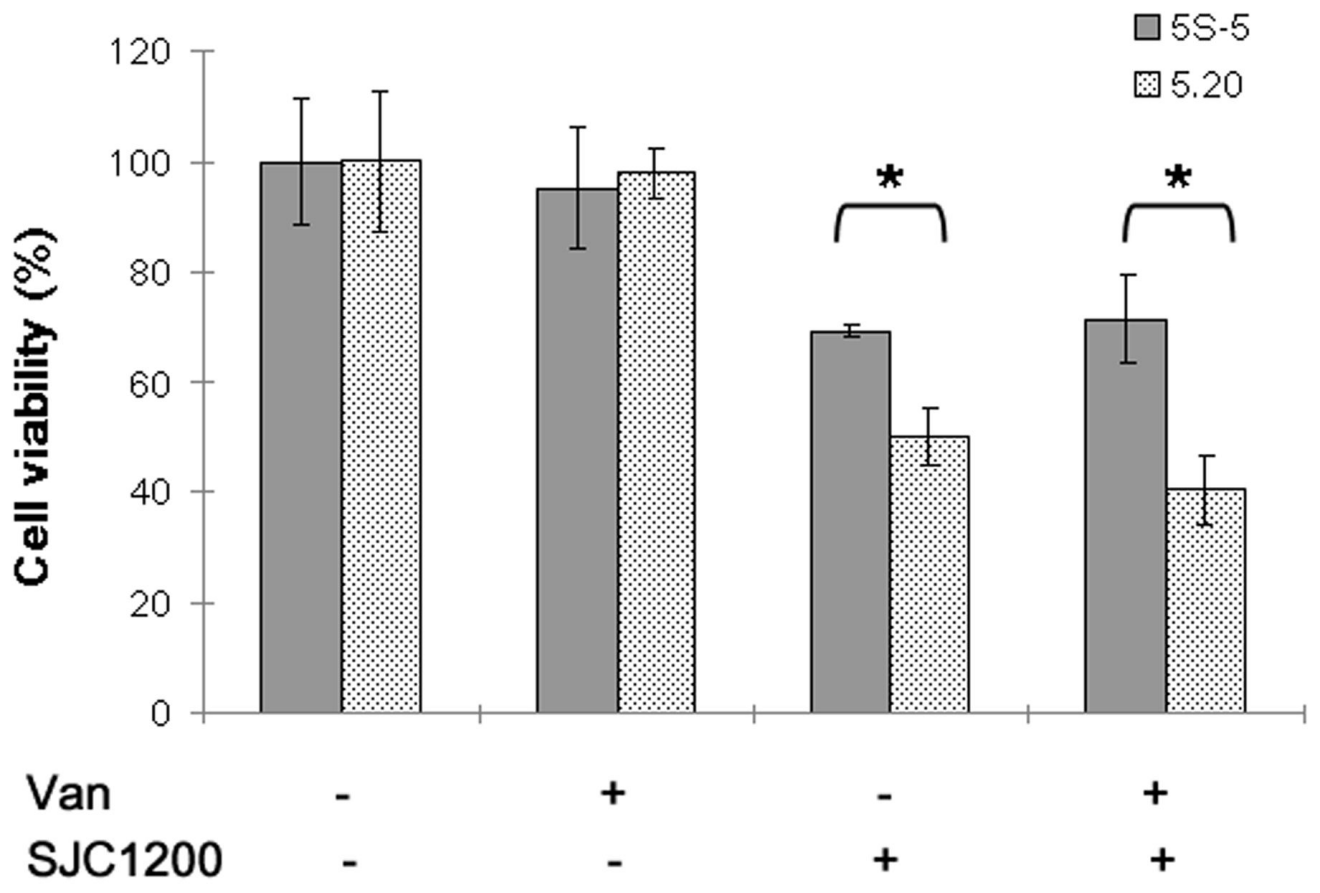
Effects of VRSA infection on cytotoxicity. Cytotoxicity toward control scramble (5S-5) or G6PD-deficient (5.20) cells that were inoculated with the VRSA strain SJC1200 (MOI=100) in the presence/absence of vancomycin (32 μg/ml) for 16 h. Cell viability was defined as 100% when cells were cultured in medium only. *P < 0.05.

To understand whether G6PD-deficient epithelial cells were less tolerant than control cells to bacterial infection, the MTT assay was employed to evaluate cell viability upon *S. aureus* infection. The results shown in Figure 1 indicate that cell viability of both A549-5S-5 (the scramble control) and A549-5.20 (G6PD-deficient) cell lines was decreased at 16 h post-infection. However, A549-5.20 cell viability (50%) was significantly less (*P* < 0.05) than that of A549-5S-5 cells (69%). 

We previously demonstrated that vancomycin could activate σ^B^ in vancomycin-resistant *Staphylococcus aureus* resulting in the enhancement of cytotoxicity [22]. The effect of vancomycin-treated VRSA infection on A549 cells and their G6PD-deficient counterparts was investigated. No additional effect was observed upon vancomycin treatment in either the scramble control or G6PD-deficient cells (Figure 1).

### Increased production of intracellular ROS in G6PD-deficient cells upon VRSA infection

**Figure 2:**
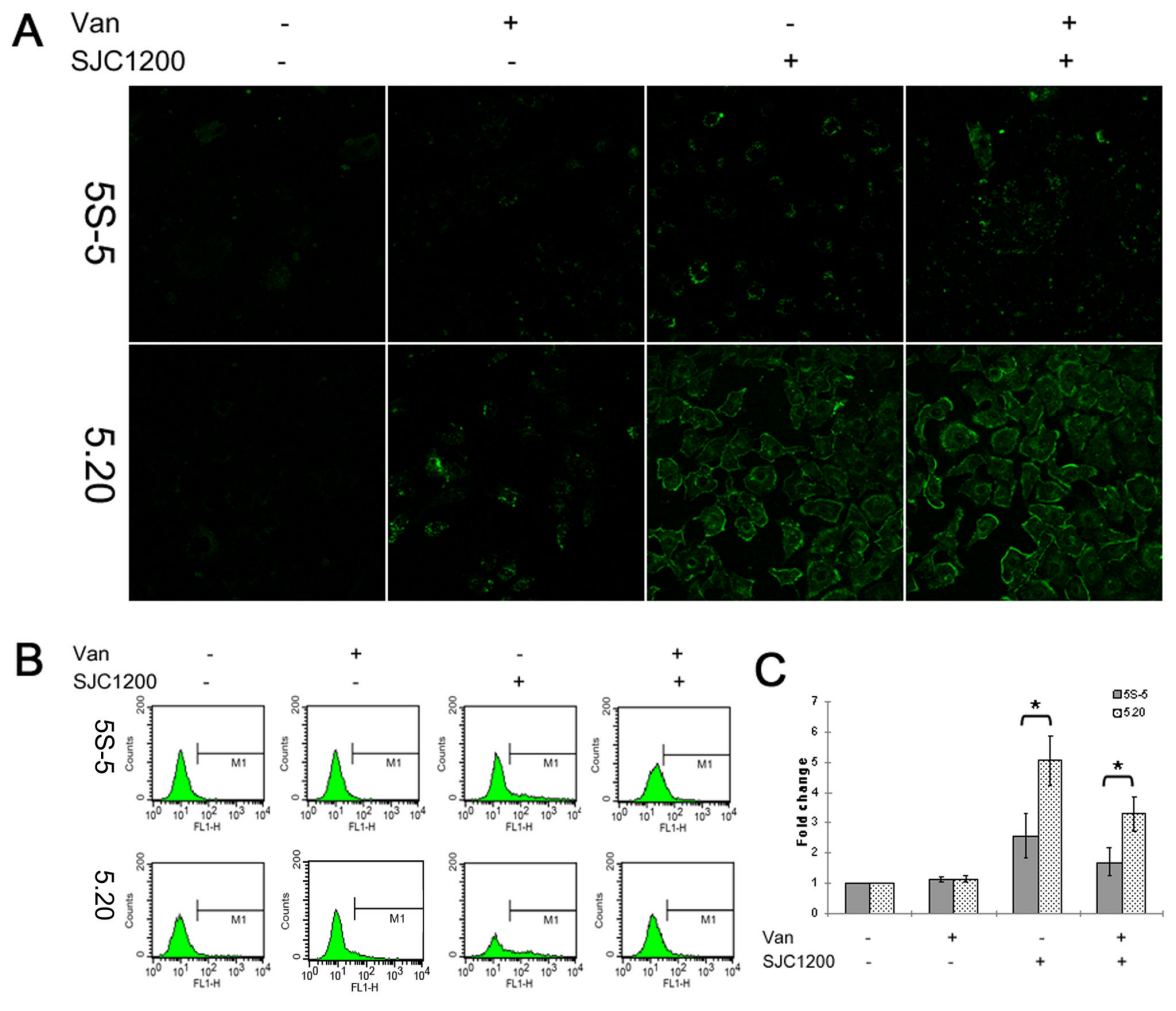
Increased production of intracellular ROS in G6PD-deficient cells upon VRSA infection. (A) At 12 h post-infection with VRSA (MOI=100), the production of intracellular ROS by control scramble or G6PD-deficient cells was observed by detecting fluorescent DCF under a fluorescence microscope. (B) ROS formation was quantified by flow cytometry, and M1 represents the percentage of ROS production. (C) Data are expressed as a fold change in the percentage increase in MFI of DCF channel relative to that of untreated cells. *P < 0.05.

We hypothesized that G6PD-deficient cells are less tolerant to oxidative stress upon bacterial infection, leading to the accumulation of more intracellular ROS when compared to the control scramble cells. This was confirmed by directly observing DCF intensity within cells by fluorescence microscopy. As shown in Figure 2A, the average level of green fluorescence intensity was higher in VRSA-infected cells than in untreated cells of both the A549-5S-5 and A549-5.20 cell lines. An even stronger fluorescence intensity was observed among VRSA-infected A549-5.20 cells than among A549-5S-5 cells. Treatment with vancomycin showed no obvious effect on the fluorescence intensity of either cell line upon VRSA infection, but a slight increase in ROS production was observed in vancomycin-treated control cells when compared to vancomycin-free cells of both cell lines. 

The production of ROS was then further quantified by determining the MFI of the DCF channel. Infection by VRSA resulted in a 2.6- and 5.0-fold increase in MFI among A549-5S-5 and A549-5.20 cells, respectively (Figure 2B and 2C). A more significant accumulation of ROS production in G6PD-deficient cells than in control scramble cells (*P* < 0.05) was observed upon bacterial infection. Vancomycin treatment of VRSA-infected cells also significantly reduced ROS accumulation in A549-5S-5 and A549-5.20 cells, and vancomycin treatment alone showed a slight but non-significant increase in ROS production in both cell lines (Figure 2B and 2C). 

To rule out reduced ROS accumulation that might be due to increased bacterial killing upon vancomycin treatment, a bacterial killing kinetic assay was performed. Slower bacterial growth was observed in the presence than in the absence of vancomycin for the first 9 hours, and no significant difference was seen for the duration of the experiment (24 h; Figure S1). In addition, at 12 h post-infection, aliquots of bacteria-infected cell culture media were removed and plated on agar, but no significant difference between the cfus of the different experimental conditions was evident (data not shown). The *van* operon-carrying plasmid (pG1546) was also found in all of the colonies, indicating that the vancomycin resistance construct was stable in SJC1200 (data not shown). 

### Enhanced apoptotic and necrotic activity in G6PD-deficient cells upon VRSA infection

**Figure 3:**
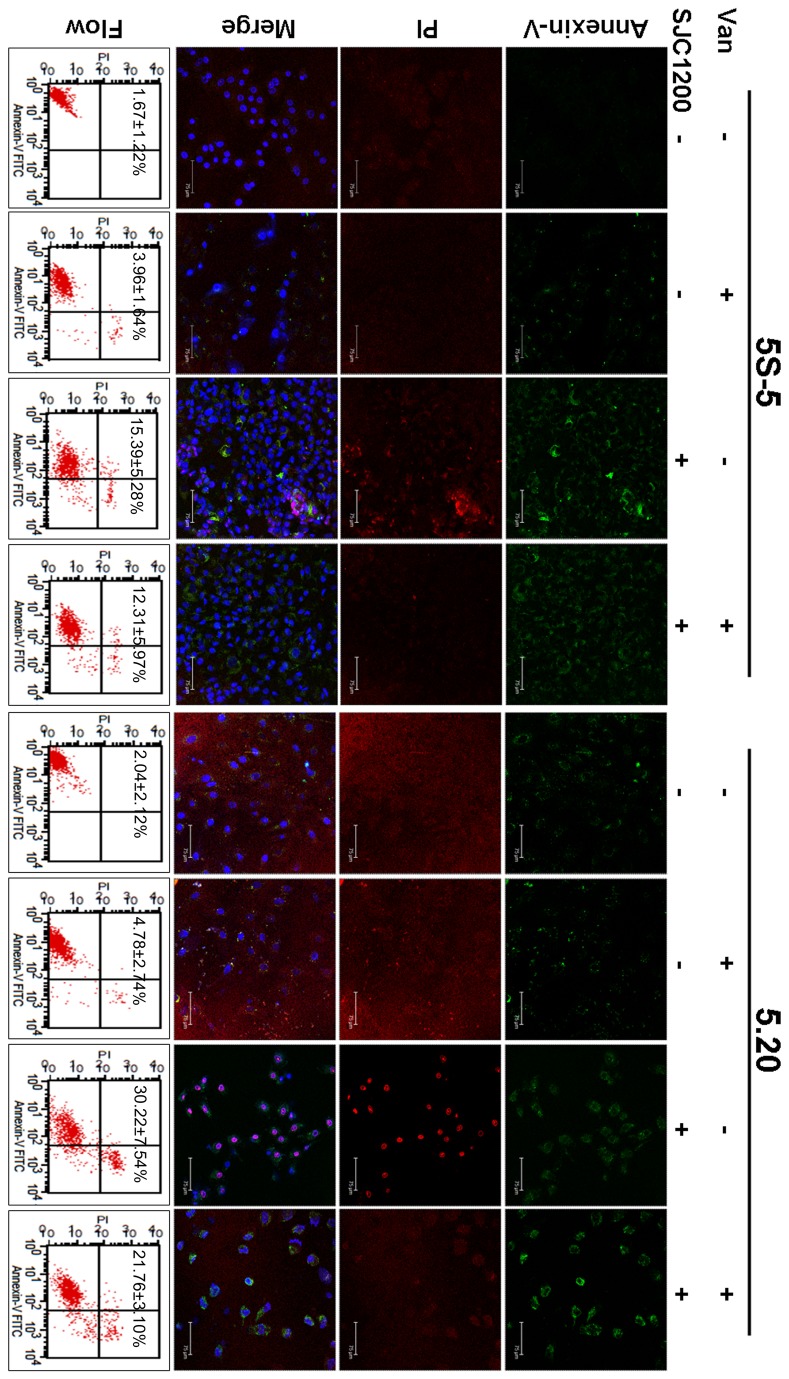
Enhanced apoptotic and necrotic activity of G6PD-deficient cells upon VRSA infection. At 16 h post-infection, cell apoptosis/necrosis was visualized by Annexin V/PI staining under a confocal fluorescence microscope. The third row represents the merge of fluorescence image of Annexin V, PI, and DAPI. The quantification assay for apoptotic activity was evaluated by flow cytometry (bottom row). Percentages of apoptotic and necrotic cells are presented as the means±sd of three separate experiments.

Whether the accumulation of more intracellular ROS resulted in stronger apoptotic activity in G6PD-deficient cells was investigated by fluorescence microscopy. The results shown in Figure 3 indicate that most of the A549-5.20 cells showed apoptosis, necrosis or both phenotypes upon VRSA infection, whereas apoptotic activity was weaker in A549-5S-5 cells under the same condition. Vancomycin treatment in VRSA-infected cells apparently reduced apoptotic activity in both A549-5S-5 and A549-5.20 cells, as detected by the proportion of PI-stained cells. Vancomycin treatment alone had no significant effect on apoptotic activity in either cell line.

The quantification assay for apoptotic activity was evaluated by flow cytometry. The percentage of apoptotic and necrotic cells increased to 15.39% in VRSA-infected A549-5S-5 cells at 16 h post-infection. The percentage was significantly higher in A549-5.20 cells (30.22%; *P* < 0.05) than in control scramble cells (Figure 3, bottom row). As observed microscopically, vancomycin treatment of VRSA-infected cells significantly reduced the percentage of apoptotic and necrotic cells to 12.31% and 21.76% in A549-5S-5 and A549-5.20 cells, respectively. We detect a slight and non-significant increase in apoptotic activity upon vancomycin treatment alone in both A549-5S-5 and A549-5.20 cells (Figure 3). 

### A higher apoptotic activity in G6PD-deficient cells through the intrinsic pathway upon VRSA infection

**Figure 4:**
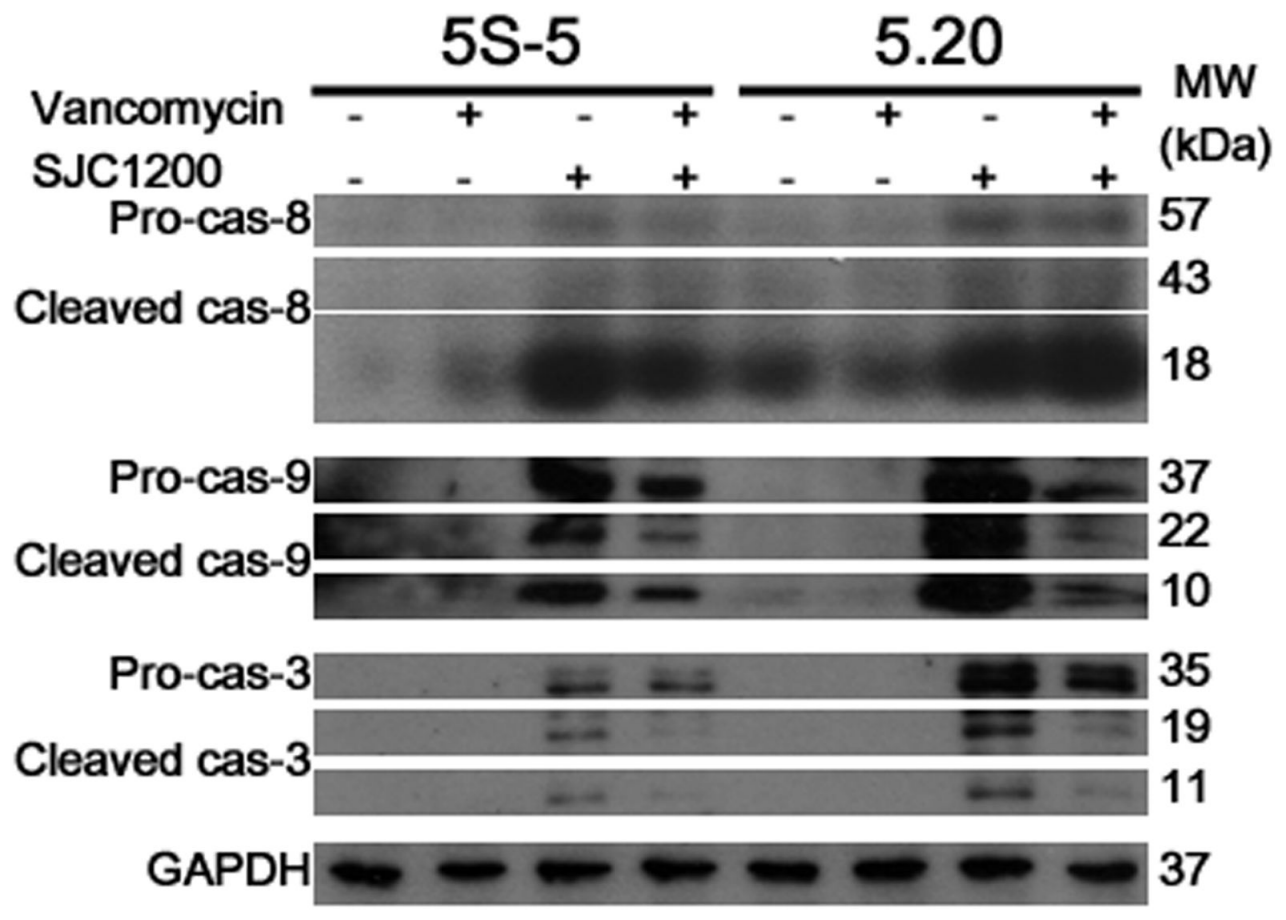
A higher apoptotic activity in G6PD-deficient cells through the intrinsic pathway upon VRSA infection. An obviously higher expression level of the intrinsic pathway initiator caspase-9 (cleaved form), as well as the downstream effector caspase-3 (cleaved form), was observed following VRSA infection in G6PD-deficient cells when compared to control cells by Western blotting analysis.

In addition to morphological markers, biochemical markers of apoptosis were detected by Western blotting analysis. Because caspases-8, -9 and -3 are located at key junctions in apoptosis pathways, their expression in A549-5S-5 cells was compared to that in A549-5.20 cells upon different treatments [27,28]. The results shown in Figure 4 indicate that a similar level of the cleaved caspase-8 (33 and 10 kDa) was observed upon VRSA/vancomycin-treated VRSA infection in both A549-5S-5 and A549-5.20 cells, suggesting a similar signal strength to trigger the extrinsic apoptotic pathway. A weak cleaved caspase-8 expression was found in bacteria-free A549-5.20 cells, implying that the G6PD-deficient cells were less tolerant to extrinsic death ligands during culture. On the other hand, VRSA infection obviously triggered higher expression of the precursor (37 kDa) and cleaved forms (22 and 10 kDa) of caspase-9, thereafter leading to higher expression levels of pro-caspase-3 (35 kDa) and cleaved caspase-3 (20 and 11 kDa), which is the effector caspase, in A549-5.20 cells than in scrambled control cells (Figure 4). The above results suggest that a stronger intrinsically apoptotic signal was triggered in G6PD-deficient cells upon bacterial infection. In addition, vancomycin treatment of VRSA-infected cells reduced the expression levels of cleaved caspase-9 and -3 in both cell lines (Figure 4). Trace or non expression of caspase-9 and -3 was observed in bacteria-free cell cultures. 

### Alpha-hemolysin plays a role in the accumulation of more ROS and enhanced apoptotic activity in G6PD-deficient cells

**Figure 5:**
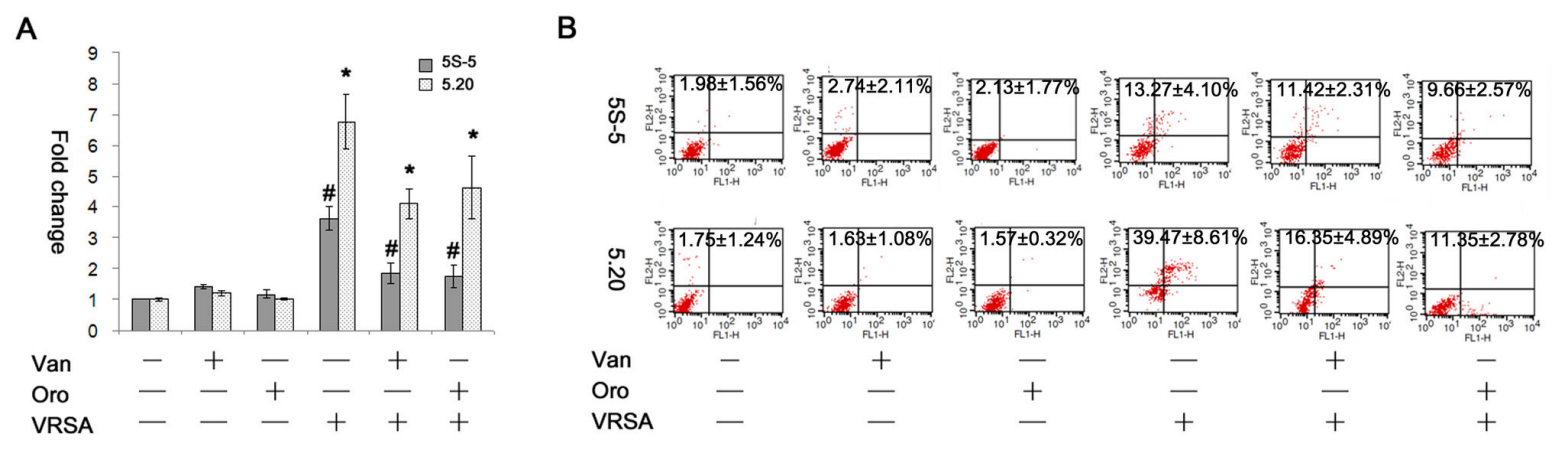
Decreased ROS production and apoptotic activity in G6PD-deficient cells upon Oroxylin A treatment. (A) At 12 h post-infection with VRSA (MOI=100), the production of intracellular ROS by control scramble or G6PD-deficient cells in the presence of Oroxylin A (2 μg/ml) or vancomycin (32 μg/ml) was quantified by determining the fold change in the percentage increase in MFI of DCF channel relative to that of untreated cells. * or #: *P* < 0.05. (B) The quantification assay for apoptotic activity was evaluated by flow cytometry, and the percentages of apoptotic and necrotic cells are presented as the means±sd of three separate experiments. Van: vancomycin, Oro: Oroxylin A, VRSA: SJC1200.

We previously showed that a decrease in *hla* (encoding α-hemolysin) expression and hemolytic activity was observed in strain SJC1200 upon vancomycin treatment [22]. To determine whether the reduced ROS accumulation and apoptotic activity, particularly in G6PD-deficient cells, was due to deceased α-hemolysin expression upon VRSA infection in the presence of vancomycin, the production of intracellular ROS and cell apoptosis when the α-hemolysin inhibitor Oroxylin A was added to the media was quantified by flow cytometry. Hemolysis was inhibited when SJC 1200 cells were inoculated on blood agar plates containing either Oroxylin A (2 μg/ml) or vancomycin (32 μg/ml) (Figure S2). Treatment with Oroxylin A or vancomycin, significantly reduced the intracellular ROS and apoptotic activity of both cell lines in the presence of infection. Oroxylin A treatment alone showed no significant effect on the accumulation of ROS or cell apoptosis (Figure 5).

## Discussion

In the present study, we demonstrate that G6PD-deficient epithelial cells were less tolerant to *S. aureus* infection through the accumulation of more ROS leading to stronger apoptotic activity. Studies on patients with G6PD deficiency have mostly focused on severe hemolytic anemia, extreme hyperbilirubinemia (jaundice), and bilirubin encephalopathy, especially in neonates [1]. As described, the effect of infection on patients with G6PD deficiency was studied mainly with regard to hemolytic anemia caused by *Plasmodium* infection, antimicrobial drugs, and viral and bacterial infections. In addition to hemolytic anemia, the enhancement of both viral infection and immune weakness (such as neutrophil dysfunction) were also addressed. Our study demonstrates a direct impact of bacterial infection on G6PD-deficient epithelial cells, a result that is in contrast to that found in blood cells. 

The lack of ROS (such as O_2_^-^) production in phagocytes (neutrophils and monocytes) may cause chronic granulomatous disease-like clinical symptoms in patients with G6PD deficiency. Such phagocyte dysfunction may lead to recurrent infections by catalase-positive microorganisms (such as staphylococci) because those bacteria could be phagocytosed but not digested [29]. On the other hand, it has been demonstrated that bacterial infection may induce oxidative stress in lymphocytes, in mammary epithelial cells, or in yeast [30–32]. Consistent with the above findings, our results indicated that intracellular ROS were generated in A549 lung carcinoma cells upon *S. aureus* infection (Figure 2). We further propose that the capacity for ROS removal is impaired in G6PD-deficient cells following infection-induced oxidative stress. As expected, the accumulation of ROS was significantly higher in G6PD-deficient cells than in control scramble cells following exposure to VRSA (Figure 2). Therefore, the impact of bacterial infection on patients with G6PD deficiency is two-fold. First is the immune weakness due to the dysfunction of phagocytes. Second is the impairment of ROS removal upon bacterial infection, as we have demonstrated in epithelial cells. 

It has been well demonstrated that apoptosis signal-regulated kinase 1 (ASK1), a member in the mitogen-activated protein kinase (MAPK) cascades, is activated upon oxidative stress, leading to cell apoptosis [33]. We demonstrated that the impairment of ROS removal in G6PD-deficient cells resulted in a higher apoptotic activity than in control scramble cells (Figure 3). The caspases, a family of cysteine proteases, play essential roles in apoptosis. The classical apoptotic machinery involves the activation of initiator caspases such as caspase-8 and -9 upon death signals, thereby cleaving the inactive pro-forms of effector caspases such as caspase-3 to activate them [27,28].The commencement of the extrinsic apoptotic pathway depends on the activation of caspase-8 through the binding of extracellular death ligands to transmembrane death receptors. In contrast, the intrinsic pathway is triggered by the release of cytochrome *c* from mitochondria, thereby activating caspase-9. Both pathways can lead to the activation of caspase-3 and other effector caspases [34]. Our results presented here indicate that expression of active caspase-9, as well as the downstream caspase-3, was much higher in G6PD-deficient cells than in control scramble cells upon VRSA infection, suggesting that mitochondrial dysfunction may be the major cause of the increase in cell apoptosis (Figure 4). A recent study demonstrated that the incubation of epithelial cells with enterotoxin isolated from *Aeromonas veronii* increased the accumulation of intracellular ROS leading to a loss of mitochondrial membrane potential and thereafter undergoing apoptosis through the mitochondrial pathway [20]. The *S. aureus* strain SJC1200 used in this study was constructed under the ATCC 12598 strain genetic background which is known to lack a variety of exotoxins except for α-hemolysin [35]. Therefore, we may propose that α-hemolysin could cause mitochondria dysfunction similar to that found in *A. veronii*. We further highlight a much worse outcome in G6PD-deficient cells upon bacterial infection. Unlike the lack of mitochondria in peripheral red blood cells (RBCs), the impact of bacterial infection on RBCs (causing hemolysis) and other cells is different in patients with G6PD deficiency. 

Our previous study demonstrated that antibiotic treatment on a drug-resistant *S. aureus* strain would appear to be an environmental stress that activates σ^B^, a stress response transcription factor [22]. In that study, vancomycin induced σ^B^ activity led to the alternation of downstream virulence genes in VRSA strains, as well as the increase in cytotoxicity to the human bronchial epithelial cells (BEAS-2B). The increased expression of bacterial binding capacity, but not the decreased expression of α-hemolysin, associated with increased σ^B^ activity, might be the key contributor to the cytotoxicity. However, similar results were not observed in this study when the A549 cell line was used, neither in G6PD knock down or in control scramble cells. It has been reported that α-hemolysin was an important mediator of cytotoxicity in A549 cells whereas it was tolerated by BEAS-2B cells [22,36]. As proposed above, α-hemolysin should be the major cause of mitochondria dysfunction through the accumulation of ROS in A549 cells and derivatives. Because the expression of α-hemolysin was decreased in SJC1200 upon vancomycin treatment, the impact of vancomycin treatment on the cytotoxicity of VRSA-infected A549 cells was not obvious in either G6PD knock down or control scramble cells (Figure 1). In fact, vancomycin treatment alleviated the VRSA infection-enhanced ROS accumulation and apoptosis in both cells even though cell survival was not improved (Figures 1-4). This phenomenon may be a result of the increased binding affinity of VRSA to the cells upon vancomycin treatment, which leads to stronger cytotoxicity and possibly bypasses the mitochondria dysfunction pathway, as we reported previously [22]. Therefore, the net impact of vancomycin treatment on A549 cell survival in the presence of infection was similar to the vancomycin-free condition. Nevertheless, we cannot rule out that the misuse of antibiotics in other drug-resistant strains will trigger the expression of other exotoxins that can cause a stronger oxidative stress in G6PD-deficient cells. Although the susceptibility of different cell types to different bacterial virulence factors is variable, we suppose patients with G6PD deficiency are less tolerant to bacterial infection. 

In conclusion, we demonstrate a cell damage mechanism in G6PD-deficient epithelial cells upon bacterial infection. Most of the cellular ROS are generated from the mitochondria, where there are a variety of mechanisms to reduce the accumulation of ROS or protect against oxidative stress [37]. The loss of mitochondrial membrane potential due to bacterial infection, possibly via *S. aureus* α-hemolysin in this case, leads to an increase in ROS production. In G6PD-deficient cells, the impaired reconversion of glutathione disulphide to reduced glutathione caused by the imbalanced NADPH/NADP^+^ redox state renders the cell unable to remove ROS effectively. Therefore, the accumulation of excess ROS results in stronger cell apoptosis through the mitochondrial pathway. One of the most important clinical concerns in treating bacterial infection in patients with G6PD deficiency is to avoid the usage of antibiotics which can cause hemolytic anemia [7]. Vancomycin treatment did not further enhance VRSA cytotoxicity toward G6PD-deficient and control scramble A549 cells, as opposed to what we had previously observed in BEAS-2B cells [22]. 

## Supporting Information

Figure S1 **Time-kill curve of the VRSA strain SJC1200.** Strain SJC1200 was incubated without (open diamond) or with vancomycin (32 μg/ml; solid circle), and the results are presented as the means±sd of the log_10_ CFU/ml from three separate experiments.
(TIF)

Figure S2 **The effects of Oroxylin A or vancomycin treatment on the hemolytic activity of SJC1200 cells.** SJC1200 cells (2×10^4^ cfu) were inoculated onto a (A) blank blood agar plate or (B) plate containing vancomycin (32 μg/ml) or (C) Oroxylin A (2 μg/ml) and incubated at 37°C overnight.
(TIF)
