# Supplementary figures and images for: Glucose-6-Phosphate Dehydrogenase (G6PD)-Deficient Epithelial Cells Are Less Tolerant to Infection by Staphylococcus aureus
Source: PLoS One. 2013 Nov 4;8(11):e79566. doi: 10.1371/journal.pone.0079566 (PMC3817128; doi:10.1371/journal.pone.0079566)

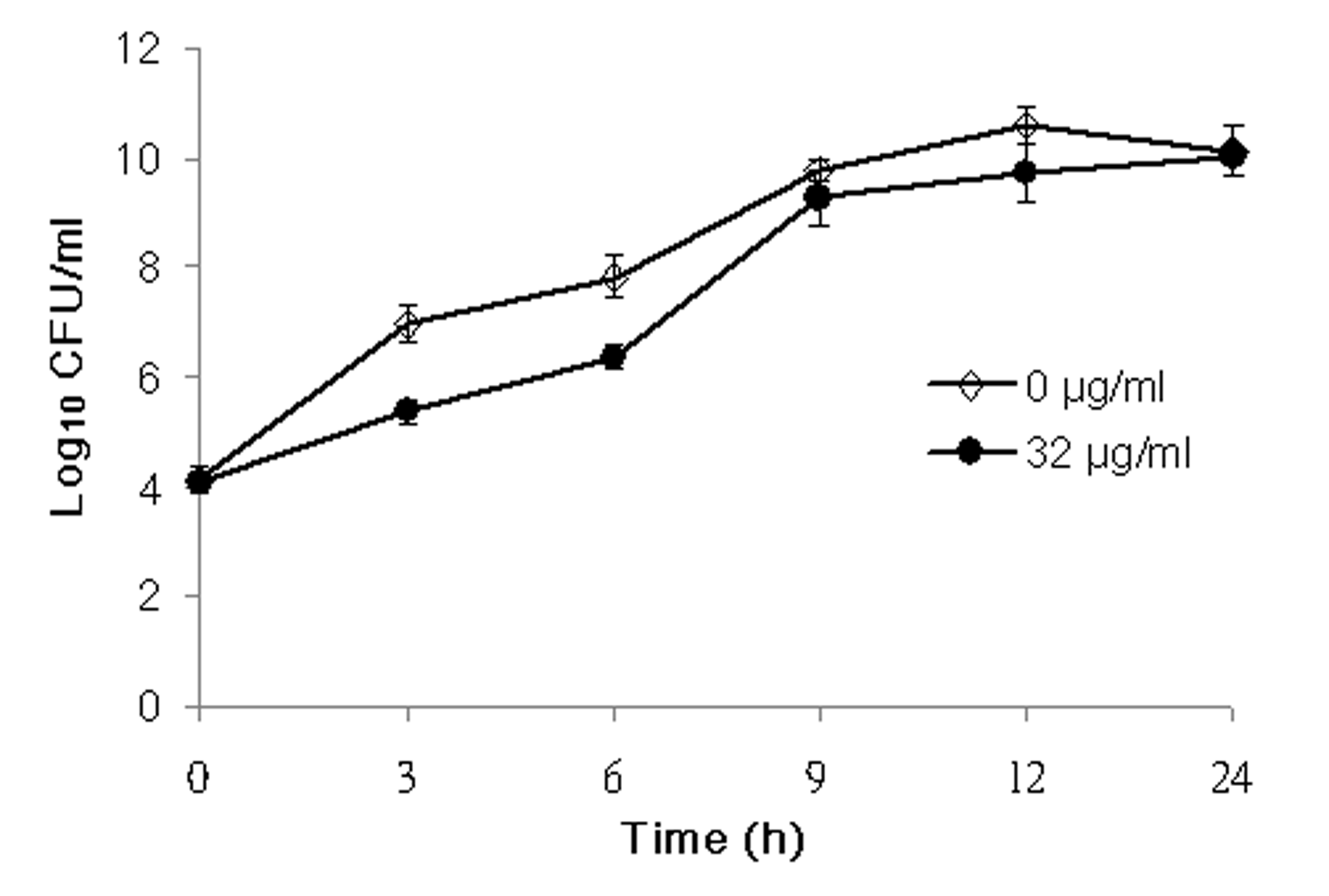

Supplement: Figure S1 — Time-kill curve of the VRSA strain SJC1200. Strain SJC1200 was incubated without (open diamond) or with vancomycin (32 μg/ml; solid circle), and the results are presented as the means±sd of the log10 CFU/ml from three separate experiments. (TIF) [file pone.0079566.s001.tif]

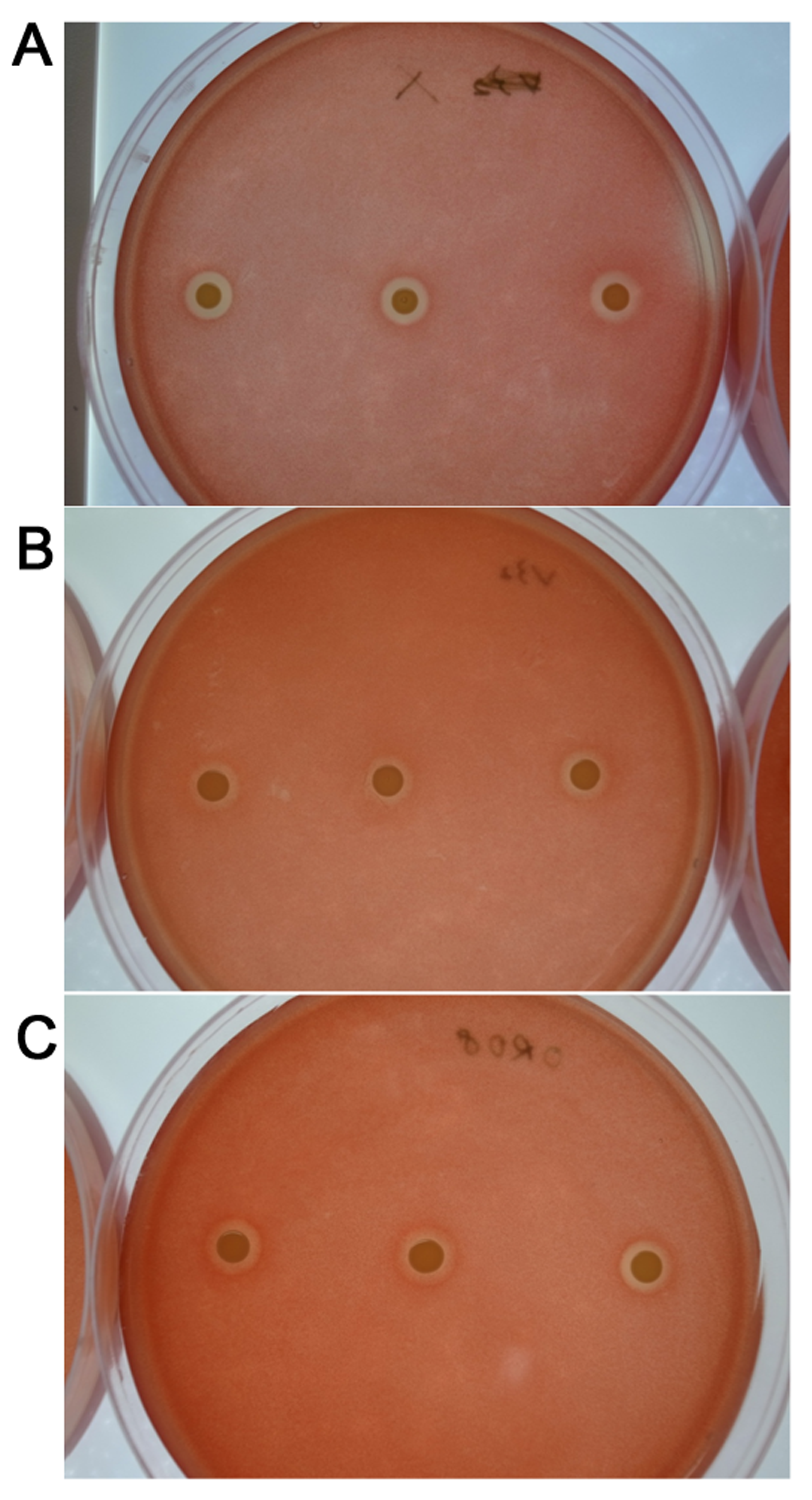

Supplement: Figure S2 — The effects of Oroxylin A or vancomycin treatment on the hemolytic activity of SJC1200 cells. SJC1200 cells (2×10^4^ cfu) were inoculated onto a (A) blank blood agar plate or (B) plate containing vancomycin (32 μg/ml) or (C) Oroxylin A (2 μg/ml) and incubated at 37°C overnight. (TIF) [file pone.0079566.s002.tif]
